# Characteristics and outcomes of diabetic patients infected by the SARS-CoV-2

**DOI:** 10.11604/pamj.2020.37.32.25192

**Published:** 2020-09-08

**Authors:** Saloua Elamari, Imane Motaib, Saad Zbiri, Karim Elaidaoui, Asmaa Chadli, Chafik Elkettani

**Affiliations:** 1 Department of Endocrinology, Diabetology, Metabolic Disease and Nutrition, Mohammed VI University of Health Sciences, Casablanca, Morocco,; 2 Laboratory of Medical Evaluation and Health Economics, International School of Public Health, Mohammed VI University of Health Sciences, Casablanca, Morocco,; 3 Department of Anesthesiology, Mohammed VI University of Health Sciences, Casablanca, Morocco

**Keywords:** Diabetes, COVID-19, SARS-CoV-2, severity

## Abstract

Diabetes is considered a risk factor for complications due to COVID-19. In order to clarify this association, we are exploring the characteristics, the clinical signs, the outcomes and death in diabetic patients with COVID-19. In this retrospective observational study we are evaluating the demographic characteristics, the comorbidities of the patients, the clinical signs of the infection, the signs of clinical severity, the biological assessment at admission, the treatment, the outcomes and the deaths of 133 patients with COVID-19, of which 25 (19,4%) had diabetes. In the compared COVID-19 patients, with and without diabetes, the patients with diabetes were older, had higher blood pressure and more cardio-vascular diseases. Severe forms were more present in diabetic patients (56% versus 27.1%). Weight loss was higher in diabetic patients (6kg versus 3kg). Biologically, diabetic patients had higher levels of C-reactive protein (28 versus 5.8mg/l), procalcitonin (0.28 versus 0,13ng/l), ferritin (501 versus 140ng/ml), lactic dehydrogenase (268 versus 226IU/l) and of D. dimer (665 versus 444μg/l). Diabetic patients required more oxygen therapy (60% versus 26.9%), more mechanical ventilation (20% versus 8.3%) and more frequent admission to the intensive care unit (60% versus 27.8%). They presented more thromboembolic complications (12% versus 9%) but there were not significant differences in the other outcomes and in death rates. The excess of morbidity and mortality due to diabetes was still not fully clarified; the role of demographic factors, the interaction of mediations with ACE-2 receptors and the role of co-morbidities will all need to be studied in order to identify the patient at risk profile, i.e. who can develop severe forms of the diseases and more outcomes. The early identification of a possible hyper inflammation could be very valuable. More attention should be paid to patients with COVID-19 with diabetes because they are at a high risk of complications.

## Introduction

SARS-CoV-2 infection was first discovered in Wuhan, China [1], in December 2019 and then spread worldwide, leading to a declaration of the pandemic by WHO in March 11, 2020. More than 13 million people were infected by the virus and there were more than 500,000 deaths by the end of June 2020 [2,3]. COVID-19 is an infection characterized by extreme contagiousness and the severity may differ from person to person. The disease can be without any symptoms or a critical illness. The critical patients present respiratory distress syndrome and/or failure of organs requiring resuscitation measures and an unfavorable prognosis [4]. Diabetes is considered a risk factor for complications due to COVID 19 [5-7]. The analysis of previous viral pandemics, SARS-CoV-1 [8,9] and influenza H1N1 [10], showed an increase in morbidity and mortality of diabetic patients. Uncontrolled diabetes is a severe factor in bacterial pneumonia [11]. Italian data shows that more than two-thirds of the COVID-19 patients who died had diabetes [12]. A Chinese study has observed an increase in viral clearance time in diabetic patients [13]. The objective of this work is to assess whether such diabetes is a risk factor for the severity and outcomes of patients with COVID-19 and try to clarify this association. In order to better describe the morbidity and mortality rates in the diabetic patients and to identify the severity factors of the clinical presentation, we need to determine the demographic characteristics of the patients, to study the specificities of the clinical presentation, to analyze the results of the biological assessments, and to compare outcomes and deaths. This could suggest new therapeutic targets and establish an effective preventive approach.

## Methods

**Study design:** this retrospective observational study evaluates the patients hospitalized for COVID-19 at the Cheikh Khalifa Al Nahyan Hospital between March 20^th^ and May 1^st^, 2020. The structure has been accredited by The Kingdom of Morocco´s Ministry of Health to welcome patients infected by SARS-CoV-2 in order to increase the supply of care in the Casablanca region, which registers 33% of confirmed cases. Seven thousand and six hundred cases have been confirmed on Moroccan territory since the declaration of the first case on March 2^nd^, 2020, including 265 deaths [3]. This study compares known diabetic patients to non-diabetic patients. Children under 15 years old and pregnant women were excluded. We evaluate the demographic characteristics, the comorbidities of the patients, the clinical signs revealing the infection, the signs of severity of the infection, the biological assessment at admission, the complications, the treatment received at the hospital and deaths. The ethics committee approval was not required for this retrospective observational study of usual patients´ data.

**Definitions:** a diagnosis of COVID-19 disease was based on the WHO interim guidance by the identification of the SARS-COV-2 viral RNA using the PCR technique in a nasopharyngeal sampling obtained by swabbing [4]. Diabetes is defined as self-reported medical history of diabetes; otherwise, newly diagnosed diabetes is based on fasting plasma glucose: glycemia higher than 2 grams per litter and classic signs of hyperglycemia during hospital stay. The HBA1C have not been carried out for all patients. Laboratory tests are performed within 24 hours of hospital admission. The severe forms and the admission in the intensive care unit is assessed according to the severity criteria defined by: respiratory rate >30 breaths per minute, severe respiratory distress or SpO2 <92% at 4l of 02, neurological or hemodynamic disorders.

**Data collecting:** for all COVID-19 patients identified, we enter the data earlier recorded into an institutional database: age, sex, weight before symptoms and in discharge from hospital, chronic medical history (diabetes, hypertension, cardio-vascular diseases, chronic kidney disease, chronic liver disease) and clinical symptoms. We also collect laboratory data such as: cells blood count, liver and renal function, reactive protein C (CRP), glycemia, ferritin, D dimer and lactic dehydrogenase (LDH).

**Statistical analyses:** the data is analyzed using SPSS (V.20.0). A value of p <0.05 was considered to be statistically significant. A study of the normality of continuous variables distribution is carried out using the Kolmogorov-Smirnov test. An independent sample t-test is applied to analyze normally distributed data and the Mann-Whitney test is applied to analyze normally undistributed data. The χ2 test and the Fisher test are applied to examine the categorical data.

## Results

**Patient demographics:** we collected the data of 133 patients hospitalized in the structure that respect the inclusion criteria. Of all the patients, 25 had diabetes (18.7%) and these were compared to 108 patients without diabetes. As shown in Table 1, diabetic patients were older (63 years versus 48 years) and had more comorbidities: high blood pressure (52% versus 21.3%) and cardiovascular disease (32% versus 10.2%). They were also more overweight or obese, however, this is statistically insignificant. There were no significant differences between the diabetic and non-diabetic patients concerning chronic renal diseases, chronic respiratory diseases, smoking, neoplastic pathologies and chronic liver diseases.

**Table 1:** demographic and clinical characteristics of the study population, with or without diabetes

	Total (n=133)	No diabetes (n=108)	Diabetes (n=25)	P-value
	Number (%)	Number (%)	Number (%)	
**Demographics**				
Age, median (IQR), years	53(36.00-64.00)	48(33.00-61.00)	63(52.00-72.00)	<0.05
Male gender	73(54.90)	58(53.70)	15(60.00)	0.36
**Comorbidities**				
High blood pressure	36(27.10)	23(21.30)	13(52.00)	<0.05
Overweight or obesity^*^	61(59.60)	47(56.00)	15(63.20)	<0.05
Cardiovascular disease	19(14.30)	11(10.20)	8(32.00)	<0.05
Kidney disease	3(2.30)	3(2.80)	0(0.00)	0.65
Liver disease	1(0.70)	1(0.09)	0(0.00)	0.80
Pulmonary disease	11(8.30)	9(8.30)	2(8.30)	0.60
Cancer	3(2.30)	2(1.90)	1(4.00)	0.40
Tobacco	7(5.20)	7(6.40)	0(0.00)	0.30

IQR: inter-quartile range; ^*^the data is missing for 28 patients

**Clinical signs of infection and the severity of diseases:** severe forms at admission were more present in diabetic patients (56% versus 27.1%). Asymptomatic forms were similar for diabetic and non-diabetic patients. Weight loss was higher in diabetic patients (6kg versus 3kg). Diabetic patients had more fever, respiratory issues, digestive issues, general issues (asthenia or/and myalgia) and headaches, but the difference was insignificant (Table 2).

**Table 2:** symptoms of COVID-19 of the study population, with or without diabetes

	Total (N=133)	No diabetes (N=108)	Diabetes (N=25)	P-value
**Clinical symptoms**				
Fever, number (%)	59(44.40)	45(42.60)	14(56.00)	0.16
Asthenia and/or myalgia, number (%)	46(46.00)	34(31.50)	12(44.00)	0.16
Headache, number (%)	21(15.80)	16(14.80)	5(20.00)	0.35
Weight loss*, median (IQR), kg	3(0.00-6.00)	3(0.00-5.00)	5.5(3.00-10.00)	<0.05
ENT, number (%)	29(21.80)	28(25.90)	1(4.00)	<0.05
Respiratory, number (%)	68(48.90)	53(49.10)	15(60.00)	0.22
Digestive, number (%)	33(24.80)	26(24.00)	7(28.00)	0.39
**Clinical forms**				
Asymptomatic, number (%)	11(8.20)	9(8.40)	2(8.00)	0.30
Serious, number (%)	45(33.80)	29(26.80)	16(64.00)	<0.05

ENT: ear, nose, throat; IQR: inter-quartile range; serious: respiratory rate >30 breaths/min; severe respiratory distress; or SpO2 ≤92% at 4l of O2, neurological or hemodynamic disorders; *the data is missing for 69 patients

**Biological assessment on admission of patients:** diabetic patients had higher levels of CRP (28 versus 5.8mg/l), procalcitonin (0.28 versus 0.13ng/l), ferritin (501 versus 140ng/ml), lactic dehydrogenase (268 versus 226 IU/l), and D dimer (665 versus 444μg/l). In diabetic patients the levels of leukocyte count and neutrophil count were higher, while the level of lymphocyte count was lower. The levels of urea nitrogen, transaminases and creatinine were higher in diabetic patients. These results remain statistically insignificant (Table 3).

**Table 3:** the blood parameters of COVID-19 of the study population, with or without diabetes

	Normal range	No diabetes (n=108)	Diabetes (n=25)	P-value
		Median (IQR)	Median (IQR)	
**Hematology**				
Hemoglobin (g/dl)	11.50-15.00	12.85(11.50-14.00)	13.00(11.80-14.20)	0.77
Platelet count (x109/L)	125.00-350.00	161.00(126.50-232.50)	173.00(130.00-230.00)	0.56
Leukocytes count (x109/L)	3.50-9.50	6.29(4.73-7.31)	6.26(4.34-7.61)	0.80
Neutrophil count (x109/L)	1.80-6.30	3.87(2.70-5.23)	4.27(2.41-6.83)	0.17
Lymphocyte (x109/L)	1.10-3.20	1.54(1.01-2.12)	1.26(0.87-1.73)	0.10
**Biochemistry**				
Random blood glucose (g/L)	0.80-1.10	1.04(0.90-1.30)	2.08(2.00-2.90)	<0.05
Aspartate aminotransferase (UI/L)	≤33.00	23.00(18.00-31.00)	30.00(21.00-41.00)	0.30
Alanine aminotransferase (UI/L)	≤33.00	19.00(14.00-33.00)	27.00(19.00-39.00)	0.50
Creatinine (mg/l)	9.00-14.00	7.50(7.04-9.40)	7.90(7.30-10.88)	0.90
Urea nitrogen (g/l)	0.10-0.55	0.19(0.19-0.30)	0.25(0.22-0.81)	0.06
Plasma protein level (g/l)	65.00-85.00	74.00(66.00-76.00)	73.00(63.00-70.00)	0.89
Lactic dehydrogenase (UI/L) ^*^	135.00-214.00	226.50(168.00-275.00)	268.00(234.00-374.00)	<0.05
**Infection**				
CRP (mg/L)	<1.00	5.80(2.00-47.00)	28(8.00-173.00)	<0.05
Procalcitonin (ng/l) ^*^	0.02-0.05	0.05(0.05-0.05)	0.05(0.05-0.10)	<0.05
Ferritin (ng/ml)^*^	15.00-150.00	140.00(41.00-302.00)	501.00(153.00-1102.00)	<0.05
**Coagulation**				
D-dimer (μg/ml)^*^	<0.50	4.44(2.75-8.77)	6.65 (5.64-19.27)	<0.05

IQR: inter-quartile range; CRP: C-reactive protein; ^*^The data is missing for 20 patients

**Therapeutic care**: diabetic patients required more oxygen therapy (60% versus 26.9%), more mechanical ventilation (20% versus 8.3%) and more frequent admission to the intensive care unit (60% versus 27.8%) (Table 4).

**Table 4:** treatment in hospital of COVID-19 of the study population, with or without diabetes

	Total (N=133) Number (%)	No diabetes (n=108) Number (%)	Diabetes (n=25) Number (%)	P-value
Admission to ICU^*^	45(33.00)	30(27.80)	15(60.00)	<0.05
Mechanical ventilation	14(10.30)	9(8.30)	5(20.00)	<0.05
Immunomodulator-therapy	5(3.80)	3(2.80)	2(8.00)	0.23
Corticotherapy	16(12.00)	10(9.30)	6(24.00)	<0.05
Hydrochloroquine + azythromicine	127(97.00)	104(96.30)	23(92.00)	0.30
Antiviral therapy	11(8.00)	9(8.30)	2(8.00)	0.65

* ICU: intensive care unit

**Outcomes and death**: diabetic patients presented more thromboembolic complications (12% versus 9%). The other outcomes, such as acute respiratory syndrome, bacterial surinfection, septic shock, acute renal failure and death, were more present in diabetic patients, but the difference was statistically insignificant (Table 5).

**Table 5:** clinical outcomes of COVID-19 of the study population, with or without diabetes

	Total (n=108)	No diabetes (n=108)	Diabetes (n=25)	P-value
	Number(%)	Number(%)	Number(%)	
ARDS ^*^	13(9.00)	9(8.30)	4(16.00)	0.20
Septic shock	9(6.00)	7(5.60)	2(8.00)	0.40
Acute renal failure	6(4.00)	4(3.70)	2(8.00)	0.36
Thrombo-embolic complications	4(3.00)	1(90)	3(12.00)	<0.05
Death	14(10.00)	10(9.30)	4(16.00)	0.20

* ARDS acute respiratory distress syndrome

## Discussion

Our study is among the first studies in the Kingdom of Morocco and North Africa to describe the clinical presentation and outcomes in Moroccan diabetic patients. Since the beginning of the COVID-19 pandemic, the question of comorbidity´s role in the course and prognosis of the infection had been raised. Diabetes is always associated with an increased predisposition to infections with a higher morbidity and mortality rate [14-16]. The purpose of this retrospective study was to describe the clinical biological characteristics and outcomes of COVID-19 in diabetic patients. The prevalence of diabetes in our series was 18.7%. The prevalence was very variable in different publications. In the Chinese series performed by Chen *et al*. [17], the prevalence was 12.1%. In the Italian series performed by Onder *et al*. [18] the prevalence was 33.9%. The prevalence was higher in the American series of Bhatjaru *et al*. [19] reaching 58%. This difference in prevalence is explained by the criteria included in the studies and the difference in diabetes prevalence of the populations. The diabetic patients in our series were older and had more comorbidities, including high blood pressure and cardiovascular diseases, than non-diabetics. Similar results were observed in the studies of Weina Guo *et al*. [20] and Yongli Yan *et al*. [21]. Diabetic COVID-19 patients had clinical signs similar to non-diabetic COVID-19 ones. They had a high proportion of symptoms of cough and dyspnea, digestive signs, fever and asthenia, although this was not statistically significant. Weina Guo *et al*. and Yongli Yan *et al*. [21] compared the clinical signs of diabetic and non-diabetic patients. Yongli Yan *et al*. [22] reported results similar to the ones in the current study. Weina *et al*. [19] reported significantly more digestive signs in the diabetic patients.

Diabetic patients lost more weight than non-diabetic patients. This is due to their admission in intensive care unit and the intensity of their inflammatory syndrome. In our work the diabetic patients presented statistically more severe forms of COVID-19 and required more admission in intensive care unit. The data suggested that patients having both COVID-19 and diabetes had more frequent combined serious or critical illness ranging from 14% to 32% in different studies [22-26]. In a study of 138 patients, Wang *et al*. [24] reported that 72% of COVID-19 patients with comorbidities, including diabetes, had to be admitted to the ICU, compared to 37% of patients without comorbidities. A meta-analysis by Roncon *et al*. demonstrated that diabetic patients with COVID-19 had a higher risk of being admitted to the intensive care unit [27]. In an analysis of 201 patients with COVID-19, Wu *et al*. [26] found that diabetic patients were more likely to develop acute respiratory distress syndrome (ARDS). The autopsy of the patients found that diabetic patients had a thickening of the pulmonary basal lamina, which may account for ARDS [23,28]. Mortality was not significantly higher in the diabetic COVID-19 patients in our study. In the Yougri *et al*. [21] series, mortality was significantly higher in the diabetics. This series only included serious or critical patients. The meta-analysis by Roncon *et al*. demonstrated that diabetic patients with COVID-19 had a higher risk of mortality [27]. Complications and death must be studied on a larger scale in order to be able to draw conclusions. In our study, we found that the serum levels of biomarkers linked to inflammation are much higher in diabetics than in non-diabetics: serum ferritin, CRP, D dimers.

This observation was noted in other studies that had compared the biological analyses of diabetic and non-diabetic patients [20,21]. A significant increase in serum ferritin indicates activation of the monocyte-macrophage system, which is a crucial part of the inflammatory storm triggered by viral replication. This finding may suggest that patients with diabetes are more likely to form the cytokine storm, which leads to severe forms of COVID-19 [5,29,30,31]. Acute inflammation activates the exogenous coagulation pathway, which leads to a more pronounced state of hypercoagulability in diabetic patients compared to non-diabetics explaining thromboembolic complications [5,30,31]. The higher procalcitonin level suggests a more frequent presence of bacterial added infection. The same result was found in the study by Yougri *et al*. [20]. The LDH level was higher in patients with diabetes compared to patients without diabetes. These abnormalities suggested that COVID-19 infections may be associated with multiple organs´ involvement in diabetic patients. However, we didn´t notice more hepatic cytolysis or acute renal failure. We did not find a difference in the blood cell counts. Lymphocytopenia [19,20] had been observed in patients with COVID-19 and correlated with prognosis. [5,30,31]. Viral infection in diabetic patients appeared to be more severe. The older the age of the patients, the more often comorbidities associated with diabetes may be a mediator of this finding. Also, chronic hyperglycemia, with a decrease in the mobilization of leukocytes and an impaired phagocytic activity, [32-34] could explain the severity of the infection.

More specific factors have been reported in the literature: increased expression of ACE-2 and furin: receptors allowing the re-entry of the virus into the intracellular, could predispose people with diabetes to a more serious SARS-CoV-2 infection [12,35,36]; altered T-cell function: the result is a deficiency in the control of viral replication and more prolonged pro-inflammatory responses [12]; increased interleukin-6: chronic inflammation accompanied by obesity and metabolic syndrome, the results were an abnormal production of cytokines and an increase in their secretion during the acute phase of infection [37,38]. Our study had the advantage of including both intentional care and conventional care patients, thus reducing the selection bias. All data were collected by primary care physicians who observed the patients directly. Our study had limitations. It was a retrospective study and the sample size was relatively limited. The diabetic and non-diabetic patients were not comparable in terms of age and comorbidities, which can lead to confusion. The excess of morbidity and mortality rates due to diabetes was still not fully clarified, it is required to study the role of demographic factors, interaction of mediations with ACE-2 receptors [19,21,22] and the role of co-morbidities, in order to identify the patient at risk profile, who can develop severe forms of the diseases and more outcomes. The early identification of a possible hyper inflammation could be very valuable [39-41].

## Conclusion

Our study found that diabetes was associated with more severe forms and more admission to the intensive care unit. Biologically it is associated with a severe inflammatory syndrome. It is essential to identify patients at risk by integrating clinical and biological parameters in order to classify them, a score can be proposed to guide the therapeutic decisions. Early therapeutic management including hospitalization, respiratory assistance and treatment with immunosuppression medicine can improve the prognosis of patients. Patients at high risk of complications should be more vigilant toward the risk of contagion and should take the necessary precautions. They must be the first candidates for vaccination. These patients should be followed during the pandemic period to adjust their treatment and control their diabetes.
